# Strategic Team Science Promotes Collaboration and Practice-Based Research at the Research Centers in Minority Institutions

**DOI:** 10.3390/ijerph20064800

**Published:** 2023-03-09

**Authors:** Yulia A. Levites Strekalova, Diana L. Kornetti, Priscilla Pemu, Tandeca King Gordon, Deepak Kumar, Michelle Brown, Shelley Spires, Elizabeth O. Ofili

**Affiliations:** 1 Clinical Translational Science Institute, Gainesville, University of Florida College of Public Health and Health Professions, Gainesville, FL 32611, USA; 2 Department of Medicine, Microbiology, Biochemistry and Immunology, and Clinical Research Center, Morehouse School of Medicine, Atlanta, GA 30310, USA; 3 Julius L. Chambers Biomedical/Biotechnology Research Institute, North Carolina Central University, Durham, NC 27707, USA; 4 Morehouse Choice Accountable Care Organization and Education System, Atlanta, GA 30315, USA; 5 Albany Area Primary Health Care, Albany, GA 31707, USA

**Keywords:** biomedical workforce, practice-based research networks, research capacity building, team science, training

## Abstract

Background. This paper reports on the implementation and evaluation of a strategy to promote collaborations and team science among investigators at the Research Centers in Minority Institutions (RCMI). The strategy presented in this paper was a hands-on workshop that allowed the application of strategic team science through structured dialogue, asset sharing, and systematic exploration of opportunities for collaboration. Methods. The workshop was attended by more than 100 participants, including RCMI and non-RCMI investigators, practice-based research network (PBRN) supplement program directors, and an NIH Institute on Minority Health and Health Disparities Program Officer. Results. A post-workshop survey was administered to collect participant feedback, assess the relevance of the workshop to the participants’ professional development goals, and gauge the applicability of the tool as a support strategy to promote collaborative research. Most of the participants acknowledged that the session met the conference objectives (95.8%), and 93.7% noted that the workshop, to a high degree, met their personal goals and objectives. During the workshop, participants shared 35 resources they were willing and able to offer for prospective collaborative projects. Conclusion. The experience reported and evaluated in this paper paves the way to understanding methods for disseminating effective strategies for inter-institutional collaborations for the sustainable growth and operation of PBRNs.

## 1. Introduction

Every facet of the scientific research enterprise, from basic laboratory research to clinical and translational research to policy formation, requires a wide range of skill sets and viewpoints combined with intellectual rigor and creativity. To increase the quality, competitiveness, and representation of diverse perspectives in US research, the NIH established the Research Centers in Minority Institutions (RCMI) program in 1985 [1]. This new funding initiative addressed congressional interest in expanding the national capability for research at institutions to educate underrepresented students in health professions or health-related sciences, to increase the equity of healthcare services, and to ensure the responsiveness of federally funded research to the needs of underserved communities. One of the success metrics of the RCMI program is increased collaboration in biomedical, clinical, and/or behavioral research that improves minority health and reduces health disparities [2].

Another national strategy that expands the capacity for health equity research is the establishment of practice-based research networks (PBRNs) [3]. Since the early 1990s, PBRNs have received support from the Agency for Health Research and Quality and other US federal agencies [4], which resulted in close to 200 networks that provide an infrastructure for embedded healthcare research, implementation of evidence-based practices, and continuous quality improvement. PBRNs have been linked to increased adoption of evidence-based research throughout the network and beyond [5], and they also benefit healthcare professionals by increasing both job and intellectual engagement and retention of healthcare care provided within what is often rote practice [5,6]. Embedded, practice-based research showed numerous documented benefits, and the continued success of PBRNs depends on the ability of academic and practice-based scholars to participate in mutually beneficial collaborative projects, accounting for the stark differences in culture and daily processes of both groups [7].

This paper supports the goal of promoting the participation of RCMI institutions in practice-based research and provides the growing RCMI-PBRN community with an evidence-based strategy to promote team science and collaboration among RCMI investigators. Specifically, this paper discusses the role and promise of collaborative research and reports on the implementation and evaluation of a strategic team science workshop.

### 1.1. Collaborative Research and Team Science

Team science is an increasingly used strategy to address the exponential growth of technology, and the increasing complexity of biomedical research challenges [8]. Team science, as defined by the National Research Council in 2015, refers to “scientific collaboration by more than one individual in an interdependent fashion, including research conducted by small teams and larger groups.” [9] The scholarship and practice of team science have gained popularity since many problems faced by both researchers and clinicians alike cross multiple disciplines and benefit from the expertise of varying perspectives due to their complexity. Inherent in team science is the concept of teamwork. Like any team, those who join in the interest of science must ensure that collaboration and communication are effective and efficient to achieving success.

A growing body of research provides information on what variables affect team development and functioning [10,11,12,13]. Effective teams are interdependent and require the interaction of individual team members to achieve shared goals [11,12,14,15]. The activities of such interdependent teams can be divided into those that represent teamwork and those that represent taskwork. To achieve the desired level of team effectiveness, research teams must be proficient in both [11]. Characteristics of effective teamwork and taskwork components have been obtained from research in teams of different sizes, from small to large, and in a variety of settings (e.g., healthcare, military, industry, academia, research) [12,13,14,16,17]. These studies make it clear that simply using a team-based approach to research projects does not in itself ensure desired results [17]. Despite the assumed benefits of collaborative research, including greater interdisciplinary connections, the presentation of diverse perspectives, and greater support for research translation, identifying prospective collaborators, forming research teams, and effectively sharing interdisciplinary knowledge, expertise, and research resources remains a challenge at both the individual investigator and institutional level [18]. Therefore, strategic planning is a vital process for teams, providing a foundation for future team activities and, as a result, team effectiveness [11]. Interventions focusing on improving team functioning and effectiveness have been found to positively impact organizational performance [17].

### 1.2. Strategic Team Science

Incentives to adopt a team science approach in research are increasingly documented in the literature [19,20,21,22]. Wood et al. [19] have reported that there is increased productivity, in terms of numbers, when publications are co-authored versus single-authored, and co-authored papers are more often cited and more likely to appear in high-impact journals [19]. To support team science efforts, higher education (IHE) has integrated interdisciplinary activities into professional development opportunities and training [19]. Exposure to such activities early in training for new researchers who are still developing their disciplinary identities, and working on expanding their depth of knowledge to identify complementary ideas and technologies, can be critical [20]. In an earlier study, Levites Strekalova et al. [20] reported guided dialogues around strategic team science that increased research self-efficacy and interdisciplinary research orientation.

The purpose of this study is to report on the content and evaluation of a team science workshop that incorporated the principles of strategic team science and aimed to promote interdisciplinary collaboration and networking in practice-based research.

## 2. Materials and Methods

### 2.1. Workshop Design

A strategic team science workshop was organized and delivered at the National Conference of Research Centers in Minority Institutions in March 2022. The session was organized to support the development of practice-based research in RCMIs and was preceded by a panel of investigators who have received recent supplemental funding from the NIH for the development of new PBRN infrastructure at their institutions. The session was attended by more than 100 participants, including investigators from RCMI and non-RCMI, supplement program directors from the practice-based research network (PBRN), and an NIH Institute of Health and Health Disparities Program Officer. The workshop was part of a larger scientific session on developing the capacity for practice-based research. The first author is an experienced facilitator with extensive experience delivering the content of the workshop to trainees, practitioners, and academic scholars.

The workshop was 60 min long and started with an overview of key concepts in strategic team science. Specifically, the facilitator introduced workshop participants to the idea that to participate in collaborations, they need to be intentional about three constructs: the power of networks [23]; equality of voice [24]; and framing questions [25]. The participants were then introduced to a strategic teaming tool, which has been previously implemented and evaluated in the context of the graduate education [20]. Here, the tool was adapted and used to promote collaborations among academic and clinical researchers, clinic managers, and research professionals.

Power of networks. PBRNs are complex social entities consisting of organizational and individual stakeholders with dynamic needs and capabilities. Although individual actors within this network may not have sufficient resources or capacity to design and implement practice-based projects, capitalizing on the power of the network helps to build collaborative relationships and interactions in these complex entities to set up infrastructure effectively and promote effective operations. This approach is critical in addressing effectiveness because no single person or organization can implement PBRNs. Therefore, traditional hierarchical models of organizational structures would not be effective in implementing PBRNs. By implementing a network structure, the PBRN can focus on the resources, expertise, and talent of a variety of organizations.

Equality of voice. Handling the complexity of PBRNs also requires equality of voice, the second concept that was discussed during the workshop. Equality of voice operationally means that participants are intentional and mindful of giving each other equal amounts of time to talk and present their ideas. It also may mean that structural elements, such as time monitoring, are consistently used to ensure that every member of a group or every participant in a dialogue has the opportunity to speak and be heard.

Framing questions. The structure of the questions emphasizes the orientation toward solutions and provides a framework for moving discussions beyond the analysis of existing health and biomedical challenges and toward identifying possible opportunities that can be brought about by collaborative action. To provide an example of the limitations of focusing on problems, one can consider a problem such as “child wellness” and ask questions such as: How do we eliminate teenage pregnancy? Or, how do we minimize bullying in schools? While those are important and significant goals in and of themselves, discussions framed in this way focus on a particular problem and may potentially be limiting for the entry of participants whose research or practice interests lie outside of those immediate problems, whereas framing questions that focus on opportunities open doors for engagement and intentional inclusion. Following the example of child health, a framing question might sound like this: What if our town were the best place to be a child? Achieving this lofty goal can include solutions to multiple problems, including teenage pregnancy and bullying in schools. However, it does not focus specifically on those problems and allows for the exchange of ideas and actions in other areas.

### 2.2. Structured Dialogue Approach

The didactic section of the workshop took about 12 min. After that, the participants broke down into small groups and were introduced to the structured dialogue technique [20]. As they began this section, the participants took turns discussing the assets that were available within the group. Whether those were their individual personal or professional assets or assets that belonged to their organizations, the participants were instructed to share only the assets they were willing and able to share. Willingness means that not all assets that are available to an organization or a professional have to be shared; some of those assets may be at capacity. An ability to share assets means that participants do not need to obtain permission to offer an asset and that the participants commit their own skill, knowledge, time, and network connections, and not the assets of somebody else in the organization. Categories of assets include the skills and knowledge of those who participate or organizations. They can represent physical assets, such as equipment, space (e.g., exam rooms or buildings and facilities), financial assets (e.g., seed capital and willingness to seek financial opportunities, such as grant writing), and time. The latter is a key asset because establishing and leading practice-based research requires a significant amount of time. Participants were encouraged to consider whether they were willing to participate in leadership activities, including being the primary point of contact for specific projects.

The second step of the discussion included combining the assets identified by the participants into new collaborative opportunities. Participants were reminded that new collaborations might not feel comfortable at first. Therefore, they were encouraged to explore the assets, ask each other questions, and be intentionally inclusive in combining the assets of multiple people. They were instructed to consider all the assets identified as available for sharing now or in the near future. They were also told to think of other potentially interested network members who were not at the table during the workshop and put it as an action item for future discussions. This activity had four active discussion groups, each identifying several assets, and then, participants were able to proceed to the discussion of opportunities.

### 2.3. Report-Back and Debriefing

Once back as a large group, participants shared examples of collaboration opportunities and discussed their possible future steps. In one of the groups, one of the participants shared knowledge in community needs assessment and health equity research, another participant offered to facilitate access to a network of community clinical providers and experience with conducting practice-based research, and yet another participant has discussed her expertise in RNA sequencing analysis. Together, the group identified and started to outline a potential collaborative opportunity to engage community clinicians in a project involving a collection and analysis of samples and data that intersect genetic and social factors of health outcomes and possible epigenetic research.

The session was concluded with a discussion of future steps, such as the identification of priorities for the groups and the necessary action steps to build on the work performed in the workshop. This conference workshop was designed as a demonstration of the method. Therefore, the workshop facilitator focused on sharing practical tips to support future collaborations. First, participants were advised to think about small actionable steps that they could commit to completing before the next meeting and gain momentum by committing to take specific steps and achieve incremental, small-scale progress. Second, participants were advised to schedule follow-up meetings for their emerging collaborative teams at the time of the current meeting to increase commitment to the nascent collaboration and facilitate follow-up for emerging leaders.

### 2.4. Evaluation Data and Analyses

Data for workshop evaluation came from three sources. First, a post-workshop evaluation survey was administered to collect participant feedback. The workshop evaluation questions were assigned to the reaction and learning levels of the Kirkpatrick evaluation model [26]. The survey items assessed the relevance of the workshop to the participants’ professional development goals and gauged the applicability of the workshop content as a support strategy to promote collaborative research. Learning levels were assessed with questions about intent to use and act upon the information presented in the session, the usefulness of the information to the participants’ professional activities of the participants, and the general learning perceived by the session participants. Second, post-workshop facilitator observation notes were used to provide a qualitative assessment of the workshop. Third, the comments and notes of resources that the participants were willing and able to share for prospective collaborative projects were recorded using an online Google Sheet during the workshop and analyzed for recurrence and uniqueness.

## 3. Results

A total of 97 participants completed a post-workshop survey. A post-workshop survey has shown that participants felt strongly engaged and satisfied with the workshop. Most of the respondents agreed that the content presented in the PBRN Awardee Workshop session was relevant and helpful. Among all respondents, 90% said the content was extremely or somewhat relevant, 95.8% of the participants stated that the content was helpful, and 93.7% said that they learned something new from the information presented in this session. Most of the participants acknowledged that the session met conference objectives (95.8%), and 93.7% noted that the session, to a high degree, met their personal goals and objectives. Regarding the future application of the content presented in this session, 85.6% of the participants said they would likely use the information in the management of embedded research activities within their clinical practice; another 12% reported that they might apply it but had not decided yet, and only 2% reported that they are unlikely to use it. After participating in this workshop, 86.6% expressed their willingness to recommend it to their colleagues, while 2% did not wish to do so, and 12.4% were undecided.

The review of open-ended survey comments on the intention to use knowledge and strategy for embedded research revealed that the session was interactive and provided actionable tools for future collaborations. Participants have also noted that the session allowed them to practice translating their knowledge and research goals into application within practice-based research. Finally, participants noted that while an online workshop allowed for interaction, an in-person format would be preferred for a more in-depth discussion of prospective collaborative projects. These comments are in agreement with the facilitator’s observations and notes. Specifically, the facilitator noted that the workshop had achieved its goal of introducing a conceptually grounded and evidence-based strategy for team science and provided a model for the implementation of similar workshops for specific projects. To move conversations from idea and resource exchange to design and implementation of practice-based projects, prospective collaborators can continue to use the structured dialogue principles presented in the workshop and supplement them with action planning and regular follow-up.

During the workshop, participants were encouraged to leave comments about research resources that they were willing and able to share with prospective research collaborators. In total, participants have recorded 35 resources on a Google Sheets document. These resources, which participants have offered as available for future collaborative projects, mostly included research expertise (e.g., skills and knowledge in health service research, experience in conducting practice-based research, optimizing clinical and research technologies) and social capital, or willingness to share access to professional networks (e.g., access to a specific network of providers, such as the Center for Maternal Health Equity).

## 4. Discussion

The annual conference of Research Centers in Minority Institutions featured a hands-on team science training session, which was reported in this publication. The event was well received by academic and clinician scholars who considered it pertinent to their professional activities. More crucially, the implementation of this workshop provided a blueprint and led to active resource sharing among prospective collaborators interested in practice-based research. This was an innovative pilot workshop that permitted the use of strategic team science through a guided dialogue approach. As the workshop evaluations showed, this format was highly effective in providing structure to the initial team development in a variety of contexts and involving scholars from various academic areas. The structured nature of this workshop and approach allows for easy replication. It also provides a sense of what each group accomplishes and how the results of the workshop could be summarized and shared. Throughout the workshop, participants were encouraged to use Google Sheets, write down the assets that they proposed to share, and note the opportunities that they were discussing. Google Sheets served as a medium for distributed cognition, storing ideas that would otherwise be shared verbally and forgotten after the workshop. Such distributed cognition and planning for future meetings also allow new collaborators to join the conversations and onboard more quickly as active members of emerging networks and discussion groups.

Conceptually, the experience reported and evaluated in this paper paves the way to understanding the methods for disseminating effective strategies for interinstitutional collaborations. Although research institutions invest in training activities and opportunities for interdisciplinary collaboration through pilot funding, the success of team science is not completely dependent on the efforts of individual researchers. Leadership engagement and participation (commitment) are critical to the success of team science collaborations and may require different levels of leadership (e.g., from various disciplines and visionary and supportive leaders) to advance future research [21]. Finally, team science is clearly incentivized in the larger research community. Specifically, there is an increased expectation of engagement in team science as a priority for funding sources, such as the National Institutes of Health’s (NIH) National Center for Advancing Translational Sciences, the International Network for the Science of Team Science, and the National Science Foundation. The existing team science literature points to increased favorability of grant proposals when publication histories demonstrate team science collaborations [19]. These incentives for team science initiatives are also driven by funding sources, such as the NIH (2018), which seek to fund those initiatives that “drive major improvements in translational efficiency and effectiveness, since what is difficult or impossible for one member of the team can be easy for a teammate with a different skill set.”

PBRNs are most often community-oriented and provide a diverse, real-world space to conduct research and identify potential research questions that arise during the delivery of patient care. However, to be sustainable, PBRNs also need access to research resources that the Clinical Translational Science Institutes and other research infrastructure entities can provide. The relationship between PBRNs and entities devoted to academic research amplifies the reach of both organizations. When partnering with PBRNs, academic researchers can increase their access to communities they would otherwise have difficulty reaching, have access to patient populations for study recruitment, and a way to move research findings into communities with which the PBRNs interact. PBRNs benefit through increased security and resources and access to a new pipeline of projects [3]. However, these collaborative relationships are likely to have varying degrees of concordance related to infrastructure access, require that the roles of each organization within the partnership are continuously clarified and evaluated, and benefit from onboarding and education to help academic partners understand how PBRNs are structured and can offer as research partners [3].

## 5. Conclusions

Participation of PBRNs in collaborative projects holds promise as an effective way to promote the translation of scientific evidence into practice. The approach presented in this paper can facilitate these relationships through structured dialogues, strategic asset sharing, and systematic exploration of opportunities for collaboration.

## Data Availability

Data inquiries should be directed to the corresponding author.
